# 2-Phenyl­acetic acid–3-{(*E*)-2-[(*E*)-pyridin-3-yl­methyl­idene]hydrazin-1-ylidenemeth­yl}pyridine (2/1)

**DOI:** 10.1107/S1600536810038390

**Published:** 2010-09-30

**Authors:** Hadi D. Arman, Trupta Kaulgud, Edward R. T. Tiekink

**Affiliations:** a Department of Chemistry, The University of Texas at San Antonio, One UTSA Circle, San Antonio, Texas 78249-0698, USA; b Department of Chemistry, University of Malaya, 50603 Kuala Lumpur, Malaysia

## Abstract

The asymmetric unit of the title 1:2 adduct, C_12_H_10_N_4_·2C_8_H_8_O_2_, comprises a single mol­ecule of 2-phenyl­acetic acid and half a mol­ecule of 3-pyridine­aldazine; the latter is completed by crystallographic inversion symmetry. In the crystal, mol­ecules are connected into a three-component aggregate *via* O—H⋯N hydrogen bonds. As the carboxyl group lies above the plane through the benzene ring to which it is attached [C—C—C—C = 62.24 (17)°] and the 4-pyridine­aldazine mol­ecule is almost planar (r.m.s. deviation of the 16 non-H atoms = 0.027 Å), the overall shape of the aggregate is that of a flattened extended chair. Layers of these aggregates are connected by C—H⋯O and C—H⋯π inter­actions and stack parallel to (220).

## Related literature

For related studies on co-crystal formation involving the isomeric *n*-pyridine­aldazines, see: Broker *et al.* (2008 ▶); Arman *et al.* (2010*a* ▶,*b* ▶).
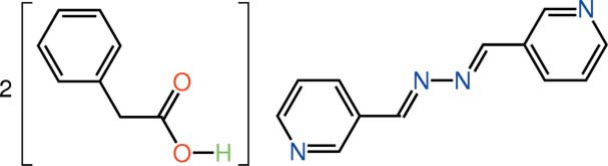


## Experimental

### 

#### Crystal data

C_12_H_10_N_4_·2C_8_H_8_O_2_
*M* *_r_* = 482.53
Triclinic, 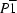

*a* = 5.511 (2) Å
*b* = 9.536 (4) Å
*c* = 12.434 (6) Å
α = 80.30 (2)°
β = 88.45 (3)°
γ = 76.46 (2)°
*V* = 626.1 (5) Å^3^
*Z* = 1
Mo *K*α radiation
μ = 0.09 mm^−1^
*T* = 98 K
0.52 × 0.32 × 0.10 mm

#### Data collection

Rigaku AFC12/SATURN724 diffractometer
Absorption correction: multi-scan (*ABSCOR*; Higashi, 1995 ▶) *T*_min_ = 0.832, *T*_max_ = 1.000
5606 measured reflections
2849 independent reflections
2578 reflections with *I* > 2σ(*I*)
*R*_int_ = 0.026

#### Refinement

*R*[*F* ^2^ > 2σ(*F* ^2^)] = 0.051
*wR*(*F* ^2^) = 0.130
*S* = 1.09
2849 reflections
166 parameters
1 restraint
H atoms treated by a mixture of independent and constrained refinement
Δρ_max_ = 0.24 e Å^−3^
Δρ_min_ = −0.21 e Å^−3^

### 

Data collection: *CrystalClear* (Molecular Structure Corporation & Rigaku, 2005 ▶); cell refinement: *CrystalClear*; data reduction: *CrystalClear*; program(s) used to solve structure: *SHELXS97* (Sheldrick, 2008 ▶); program(s) used to refine structure: *SHELXL97* (Sheldrick, 2008 ▶); molecular graphics: *ORTEP-3* (Farrugia, 1997 ▶) and *DIAMOND* (Brandenburg, 2006 ▶); software used to prepare material for publication: *publCIF* (Westrip, 2010 ▶).

## Supplementary Material

Crystal structure: contains datablocks global, I. DOI: 10.1107/S1600536810038390/hb5648sup1.cif

Structure factors: contains datablocks I. DOI: 10.1107/S1600536810038390/hb5648Isup2.hkl

Additional supplementary materials:  crystallographic information; 3D view; checkCIF report

## Figures and Tables

**Table 1:** Hydrogen-bond geometry (Å, °) *Cg*1 is the centroid of the C3–C8 ring.

*D*—H⋯*A*	*D*—H	H⋯*A*	*D*⋯*A*	*D*—H⋯*A*
O1—H1*o*⋯N1^i^	0.85 (2)	1.84 (2)	2.689 (2)	176 (2)
C8—H8⋯O2^ii^	0.95	2.47	3.398 (2)	166
C10—H10⋯O2^iii^	0.95	2.57	3.277 (2)	132
C10—H10⋯*Cg*1^iii^	0.95	2.89	3.627 (2)	135

Symmetry codes: (i) 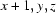; (ii) 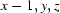; (iii) 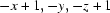.

**Fig. 1.**
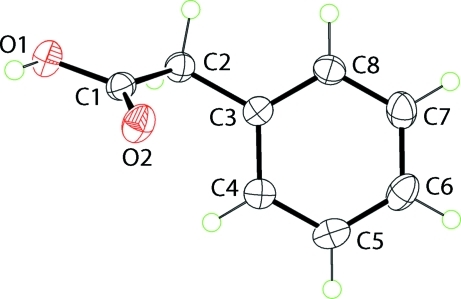
Molecular structure of 2-phenylacetic acid found in co-crystal (I) showing displacement ellipsoids at the 50% probability level

**Fig. 2.**
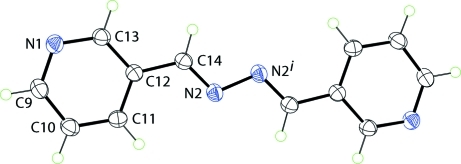
Molecular structure of 3-pyridinealdazine found in co-crystal (I) showing displacement ellipsoids at the 50% probability level. The molecule is disposed about a centre of inversion with i = 1 - x, 1 - y, -z.

**Fig. 3.**
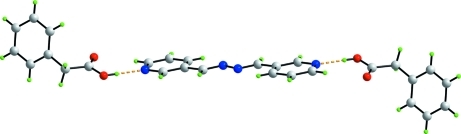
The three component aggregate in (I) highlighting the extended chair conformation. The O—H···N hydrogen bonds are shown as orange dashed lines.

**Fig. 4.**
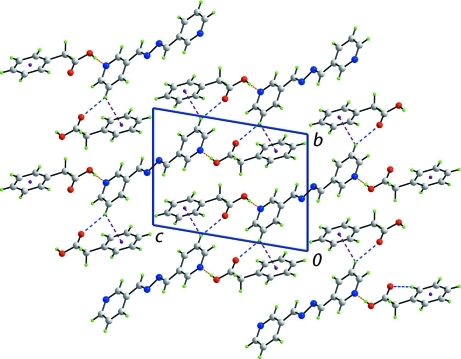
A view in projection down the a axis highlighting the stacking of layers in co-crystal (I) mediated by O—H···N, C—H···O and C—H···π interactions shown as orange, blue and purple dashed lines, respectively.

## supplementary crystallographic information

### Comment

As a continuation of studies into the phenomenon of co-crystallization of the isomeric n-pyridinealdazines (Broker *et al.*, 2008; Arman *et al.*, 2010*a*; Arman *et al.*, 2010*b*), the co-crystallization of 2-phenylacetic acid and 3-pyridinealdazine was investigated. This lead to the isolation of the title 2/1 co-crystal, (I).

The asymmetric unit in (I) comprises a molecule of 2-phenylacetic acid, Fig. 1, and half a molecule of 3-pyridinealdazine, with the latter disposed about a centre of inversion, Fig. 2. The constituents of (I) are connected by O—H···N hydrogen bonds, Table 1, to generate a centrosymmetric three component aggregate, Fig. 3. The 2-phenylacetic acid molecule is non-planar as seen in the value of the C1—C2—C3—C4 torsion angle of 62.24 (17) °. By contrast, the 4-pyridinealdazine molecule is planar with the r.m.s. deviation of the 16 non-hydrogen atoms from their least-squares plane being 0.027 Å. Hence, the three component aggregate has the shape of a flattened extended chair. The structure of co-crystal (I) resembles closely that with 4-pyridinealdazine (Arman *et al.*, 2010*b*) but the structures are not isomorphous.

In the crystal packing, the three component aggregates pack into layers parallel to (022) being connected by C—H···O and C—H···π contacts, Fig. 4 and Table 1.

### Experimental

Golden prisms of (I) were isolated from the 2:1 co-crystallization of 2-phenylacetic acid (Sigma Aldrich) and 3-[(1*E*)-[(*E*)-2-(pyridin-3-ylmethylidene)hydrazin-1-ylidene]methyl]pyridine (Sigma Aldrich) in tetrahydrofuran, m. pt. 370–373 K.

IR assignment (cm^-1^): 2923 ν(C—H); 2444 ν(O—H); 1704 ν(C=O); 1628 ν(C=N); 1498, 1455, 1410 ν(C–C aromatic); 1346, 1307 ν(C—N); 819, 746 δ(C—H).

### Refinement

C-bound H-atoms were placed in calculated positions (C–H 0.95–0.99 Å) and were included in the refinement in the riding model approximation with *U*_iso_(H) set to 1.2*U*_eq_(C). The O-bound H-atom was located in a difference Fourier map and was refined with a distance restraint of O–H 0.84±0.01 Å, and with *U*_iso_(H) = 1.5*U*_eq_(O).

### Crystal data

**Table:**

C_12_H_10_N_4_·2C_8_H_8_O_2_	*Z* = 1
*M**_r_* = 482.53	*F*(000) = 254
Triclinic, *P*1	*D*_x_ = 1.280 Mg m^−^^3^
Hall symbol: -P 1	Mo *K*α radiation, λ = 0.71073 Å
*a* = 5.511 (2) Å	Cell parameters from 2727 reflections
*b* = 9.536 (4) Å	θ = 2.6–40.1°
*c* = 12.434 (6) Å	µ = 0.09 mm^−^^1^
α = 80.30 (2)°	*T* = 98 K
β = 88.45 (3)°	Prism, gold
γ = 76.46 (2)°	0.52 × 0.32 × 0.10 mm
*V* = 626.1 (5) Å^3^	

### Data collection

**Table:**

Rigaku AFC12K/SATURN724 diffractometer	2849 independent reflections
Radiation source: fine-focus sealed tube	2578 reflections with *I* > 2σ(*I*)
graphite	*R*_int_ = 0.026
ω scans	θ_max_ = 27.5°, θ_min_ = 2.6°
Absorption correction: multi-scan (*ABSCOR*; Higashi, 1995)	*h* = −6→7
*T*_min_ = 0.832, *T*_max_ = 1.000	*k* = −11→12
5606 measured reflections	*l* = −16→16

### Refinement

**Table:**

Refinement on *F*^2^	Primary atom site location: structure-invariant direct methods
Least-squares matrix: full	Secondary atom site location: difference Fourier map
*R*[*F*^2^ > 2σ(*F*^2^)] = 0.051	Hydrogen site location: inferred from neighbouring sites
*wR*(*F*^2^) = 0.130	H atoms treated by a mixture of independent and constrained refinement
*S* = 1.09	*w* = 1/[σ^2^(*F*_o_^2^) + (0.0606*P*)^2^ + 0.17*P*] where *P* = (*F*_o_^2^ + 2*F*_c_^2^)/3
2849 reflections	(Δ/σ)_max_ = 0.001
166 parameters	Δρ_max_ = 0.24 e Å^−^^3^
1 restraint	Δρ_min_ = −0.21 e Å^−^^3^

### Special details

**Table:**

Geometry. All s.u.'s (except the s.u. in the dihedral angle between two l.s. planes) are estimated using the full covariance matrix. The cell s.u.'s are taken into account individually in the estimation of s.u.'s in distances, angles and torsion angles; correlations between s.u.'s in cell parameters are only used when they are defined by crystal symmetry. An approximate (isotropic) treatment of cell s.u.'s is used for estimating s.u.'s involving l.s. planes.
Refinement. Refinement of *F*^2^ against ALL reflections. The weighted *R*-factor *wR* and goodness of fit *S* are based on *F*^2^, conventional *R*-factors *R* are based on *F*, with *F* set to zero for negative *F*^2^. The threshold expression of *F*^2^ > 2σ(*F*^2^) is used only for calculating *R*-factors(gt) *etc*. and is not relevant to the choice of reflections for refinement. *R*-factors based on *F*^2^ are statistically about twice as large as those based on *F*, and *R*- factors based on ALL data will be even larger.

### Fractional atomic coordinates and isotropic or equivalent isotropic displacement parameters (Å^2^)

**Table:**

	*x*	*y*	*z*	*U*_iso_*/*U*_eq_	
O1	0.4813 (2)	0.37678 (11)	0.41019 (8)	0.0306 (3)	
H1o	0.616 (2)	0.346 (2)	0.3774 (15)	0.046*	
O2	0.65187 (19)	0.18266 (12)	0.53456 (8)	0.0327 (3)	
C1	0.4823 (3)	0.28746 (15)	0.50429 (11)	0.0244 (3)	
C2	0.2466 (2)	0.33170 (15)	0.56825 (11)	0.0260 (3)	
H2A	0.2104	0.4387	0.5669	0.031*	
H2B	0.1059	0.3105	0.5311	0.031*	
C3	0.2583 (2)	0.25628 (14)	0.68565 (11)	0.0233 (3)	
C4	0.4322 (3)	0.27578 (15)	0.75870 (11)	0.0260 (3)	
H4	0.5476	0.3337	0.7336	0.031*	
C5	0.4367 (3)	0.21091 (16)	0.86760 (12)	0.0301 (3)	
H5	0.5550	0.2247	0.9166	0.036*	
C6	0.2690 (3)	0.12602 (18)	0.90514 (12)	0.0339 (3)	
H6	0.2706	0.0829	0.9799	0.041*	
C7	0.0988 (3)	0.10450 (18)	0.83273 (13)	0.0347 (4)	
H7	−0.0142	0.0450	0.8577	0.042*	
C8	0.0934 (2)	0.16981 (16)	0.72377 (12)	0.0284 (3)	
H8	−0.0244	0.1551	0.6749	0.034*	
N1	−0.0907 (2)	0.29342 (13)	0.30452 (10)	0.0282 (3)	
N2	0.4880 (2)	0.43938 (12)	0.03906 (9)	0.0258 (3)	
C9	0.0632 (3)	0.16205 (16)	0.33502 (12)	0.0296 (3)	
H9	0.0136	0.0960	0.3926	0.035*	
C10	0.2924 (3)	0.11778 (16)	0.28634 (12)	0.0305 (3)	
H10	0.3954	0.0231	0.3097	0.037*	
C11	0.3679 (3)	0.21366 (15)	0.20351 (12)	0.0276 (3)	
H11	0.5233	0.1859	0.1687	0.033*	
C12	0.2118 (3)	0.35235 (15)	0.17169 (11)	0.0241 (3)	
C13	−0.0167 (3)	0.38574 (15)	0.22392 (11)	0.0266 (3)	
H13	−0.1254	0.4788	0.2011	0.032*	
C14	0.2776 (3)	0.46341 (15)	0.08762 (11)	0.0253 (3)	
H14	0.1624	0.5551	0.0685	0.030*	

### Atomic displacement parameters (Å^2^)

**Table:**

	*U*^11^	*U*^22^	*U*^33^	*U*^12^	*U*^13^	*U*^23^
O1	0.0334 (6)	0.0296 (5)	0.0252 (5)	−0.0036 (4)	0.0036 (4)	−0.0007 (4)
O2	0.0301 (5)	0.0331 (6)	0.0281 (5)	0.0021 (4)	0.0024 (4)	0.0004 (4)
C1	0.0267 (6)	0.0245 (6)	0.0224 (6)	−0.0067 (5)	−0.0020 (5)	−0.0038 (5)
C2	0.0231 (6)	0.0258 (7)	0.0265 (7)	−0.0022 (5)	−0.0011 (5)	−0.0019 (5)
C3	0.0214 (6)	0.0223 (6)	0.0242 (7)	0.0001 (5)	0.0008 (5)	−0.0054 (5)
C4	0.0254 (6)	0.0227 (6)	0.0293 (7)	−0.0025 (5)	−0.0006 (5)	−0.0062 (5)
C5	0.0280 (7)	0.0319 (7)	0.0281 (7)	0.0025 (6)	−0.0030 (5)	−0.0114 (6)
C6	0.0312 (7)	0.0391 (8)	0.0242 (7)	0.0028 (6)	0.0036 (5)	−0.0013 (6)
C7	0.0255 (7)	0.0376 (8)	0.0366 (8)	−0.0053 (6)	0.0057 (6)	0.0019 (6)
C8	0.0212 (6)	0.0315 (7)	0.0314 (7)	−0.0044 (5)	−0.0010 (5)	−0.0043 (6)
N1	0.0316 (6)	0.0279 (6)	0.0253 (6)	−0.0074 (5)	0.0043 (5)	−0.0051 (5)
N2	0.0309 (6)	0.0236 (6)	0.0229 (6)	−0.0070 (5)	0.0021 (4)	−0.0030 (5)
C9	0.0374 (8)	0.0241 (7)	0.0276 (7)	−0.0091 (6)	0.0017 (6)	−0.0029 (5)
C10	0.0354 (8)	0.0224 (7)	0.0318 (7)	−0.0037 (6)	0.0017 (6)	−0.0039 (6)
C11	0.0281 (7)	0.0253 (7)	0.0290 (7)	−0.0039 (5)	0.0024 (5)	−0.0070 (5)
C12	0.0275 (7)	0.0227 (6)	0.0224 (6)	−0.0056 (5)	0.0006 (5)	−0.0052 (5)
C13	0.0290 (7)	0.0242 (7)	0.0250 (7)	−0.0030 (5)	0.0012 (5)	−0.0042 (5)
C14	0.0280 (7)	0.0229 (6)	0.0244 (6)	−0.0042 (5)	−0.0005 (5)	−0.0045 (5)

### Geometric parameters (Å, °)

**Table:**

O1—C1	1.3254 (17)	C7—H7	0.9500
O1—H1o	0.853 (9)	C8—H8	0.9500
O2—C1	1.2120 (17)	N1—C9	1.3389 (19)
C1—C2	1.516 (2)	N1—C13	1.3397 (18)
C2—C3	1.5105 (19)	N2—C14	1.2832 (19)
C2—H2A	0.9900	N2—N2^i^	1.408 (2)
C2—H2B	0.9900	C9—C10	1.391 (2)
C3—C8	1.388 (2)	C9—H9	0.9500
C3—C4	1.4019 (19)	C10—C11	1.381 (2)
C4—C5	1.389 (2)	C10—H10	0.9500
C4—H4	0.9500	C11—C12	1.398 (2)
C5—C6	1.388 (2)	C11—H11	0.9500
C5—H5	0.9500	C12—C13	1.395 (2)
C6—C7	1.390 (2)	C12—C14	1.4602 (19)
C6—H6	0.9500	C13—H13	0.9500
C7—C8	1.390 (2)	C14—H14	0.9500
			
C1—O1—H1O	107.8 (14)	C8—C7—H7	119.9
O2—C1—O1	123.54 (13)	C3—C8—C7	120.66 (14)
O2—C1—C2	124.48 (13)	C3—C8—H8	119.7
O1—C1—C2	111.98 (12)	C7—C8—H8	119.7
C3—C2—C1	114.69 (11)	C9—N1—C13	117.66 (13)
C3—C2—H2A	108.6	C14—N2—N2^i^	111.72 (14)
C1—C2—H2A	108.6	N1—C9—C10	123.08 (13)
C3—C2—H2B	108.6	N1—C9—H9	118.5
C1—C2—H2B	108.6	C10—C9—H9	118.5
H2A—C2—H2B	107.6	C11—C10—C9	118.95 (13)
C8—C3—C4	118.88 (13)	C11—C10—H10	120.5
C8—C3—C2	120.74 (12)	C9—C10—H10	120.5
C4—C3—C2	120.37 (13)	C10—C11—C12	118.88 (13)
C5—C4—C3	120.36 (14)	C10—C11—H11	120.6
C5—C4—H4	119.8	C12—C11—H11	120.6
C3—C4—H4	119.8	C13—C12—C11	117.99 (13)
C6—C5—C4	120.29 (14)	C13—C12—C14	118.80 (12)
C6—C5—H5	119.9	C11—C12—C14	123.21 (13)
C4—C5—H5	119.9	N1—C13—C12	123.42 (13)
C5—C6—C7	119.58 (14)	N1—C13—H13	118.3
C5—C6—H6	120.2	C12—C13—H13	118.3
C7—C6—H6	120.2	N2—C14—C12	121.22 (13)
C6—C7—C8	120.21 (15)	N2—C14—H14	119.4
C6—C7—H7	119.9	C12—C14—H14	119.4
			
O2—C1—C2—C3	13.2 (2)	C13—N1—C9—C10	−0.6 (2)
O1—C1—C2—C3	−167.21 (12)	N1—C9—C10—C11	0.8 (2)
C1—C2—C3—C8	−119.47 (14)	C9—C10—C11—C12	0.3 (2)
C1—C2—C3—C4	62.24 (17)	C10—C11—C12—C13	−1.5 (2)
C8—C3—C4—C5	−0.8 (2)	C10—C11—C12—C14	177.93 (13)
C2—C3—C4—C5	177.55 (12)	C9—N1—C13—C12	−0.7 (2)
C3—C4—C5—C6	0.0 (2)	C11—C12—C13—N1	1.8 (2)
C4—C5—C6—C7	0.9 (2)	C14—C12—C13—N1	−177.69 (13)
C5—C6—C7—C8	−1.2 (2)	N2^i^—N2—C14—C12	179.76 (13)
C4—C3—C8—C7	0.5 (2)	C13—C12—C14—N2	178.28 (13)
C2—C3—C8—C7	−177.80 (13)	C11—C12—C14—N2	−1.1 (2)
C6—C7—C8—C3	0.5 (2)		

Symmetry codes: (i) −*x*+1, −*y*+1, −*z*.

### Hydrogen-bond geometry (Å, °)

**Table:**

Cg1 is the centroid of the C3–C8 ring.

**Table:**

*D*—H···*A*	*D*—H	H···*A*	*D*···*A*	*D*—H···*A*
O1—H1o···N1^ii^	0.85 (2)	1.84 (2)	2.689 (2)	176 (2)
C8—H8···O2^iii^	0.95	2.47	3.398 (2)	166
C10—H10···O2^iv^	0.95	2.57	3.277 (2)	132
C10—H10···Cg1^iv^	0.95	2.89	3.627 (2)	135

Symmetry codes: (ii) *x*+1, *y*, *z*; (iii) *x*−1, *y*, *z*; (iv) −*x*+1, −*y*, −*z*+1.
